# Global Challenges vs. the Need for Regional Performance Models under the Present Pandemic Crisis

**DOI:** 10.3390/ijerph181910254

**Published:** 2021-09-29

**Authors:** Romeo-Victor Ionescu, Monica Laura Zlati, Valentin Marian Antohi

**Affiliations:** 1 Administrative Sciences and Regional Studies, Faculty of Juridical, Social and Political Sciences, Dunarea de Jos University, 111th, Domneasca Street, 800201 Galati, Romania; ionescu_v_romeo@yahoo.com; 2 Accounting, Faculty of Economics, Administration and Business, Stefan cel Mare University, Universitatii Street, no. 13, 720229 Suceava, Romania; sorici.monica@usm.ro; 3 Business Administration, Faculty of Economics and Business Administration, Dunarea de Jos University, Nicolae Balcescu Street, no. 59-61, 800001 Galati, Romania; 4 Finance, Accounting and Economic Theory, Faculty of Economic Sciences and Business Administration, Transylvania University, Colina Universitatii Street, no. 1, 500036 Brasov, Romania

**Keywords:** European administrative capacity, econometric model, European regional development, COVID-19, population migration, economic crisis

## Abstract

The present study uses the analysis of the EU’s regional performance structure based on clusters to test the versatility of the regional administrative capacity in relation to three disruptive global phenomena: the economic crisis, the coronavirus epidemic and the phenomenon of refugee migration to Europe. We defined a regional performance model based on maintaining sustainability indicators in the 240 EU regions. The objectives of the study are aimed primarily at a structured assessment of regional administrative capacity in the initial version, based on statistical indicators, and in the current version, after the outbreak of the pandemic, based on quantifying the impact of the disturbing factors. Secondly, the objectives of the study are to evaluate the reaction of the administrative units according to their ability to respond to the economic problems in the region, in the sense of improving the performance of the regional economies. The methods used in this paper will be empirical (the study of the specialized literature), analytical and will contain econometric modelling and statistical processing of the data. The results of the study will allow the identification of the necessary traits to train a leader in regional performance, traits that will be useful to European decision makers in adjusting the EU regional policy. Moreover, the need to redefine the EU in terms of performance will be substantiated once again. The study is current and is based on the latest Eurostat information, pertinent tables and diagrams.

## 1. General Approach

The current context is an atypical one, in which disturbing factors are manifested both from the social and economic point of view. From the social point of view, regional development is directly influenced by the spread of the new virus (COVID-19), whose effects have manifested in all EU Member States. To this end, we have built a statistical database for the period 2006–2002, using Eurostat information [1].

From the regional point of view, the evolution in the most-affected Member States reflects that the regional character is typical of this disturbing phenomenon. Additionally, the regional specificity of the spread of the disease is manifested in Romania. 

Overall, it is noted that three EU-representative economies are placed in the top 12 worldwide in terms of total COVID cases’ impact in September 2021: France (6th place), Spain (11th place) and Italy (12th place). The UK has left the EU, but is ranked 4th overall. All European countries are currently facing social and economic crises amid dissent over anti-counterfeiting protective measures [2].

From the economic point of view, the direct impact estimated by the national statistical systems at the level of the four above analysed countries consists of a decrease in GDP.

In this context, the population from the other EU Member States established in areas strongly affected by COVID-19, such as France, Italy or Germany, had the possibility to return massively to their countries of origin, favouring the spread of the virus and generating social problems and economic difficulties for their country of origin. In addition to the restriction of the health system and the increasing need for equipment, medicines and medical staff compared to the planned need, this phenomenon had to provide measures to quarantine the population that returned to the country, as well as to ensure the social protection of the population that lost their places of work during the pandemic. This migration phenomenon is strictly induced by the global pandemic, but Europe is also facing another migration phenomenon of the African, Arab and East Asian populations, which raises issues for European forums regarding ensuring social protection and integrating migrants into a system already incapacitated by the outbreak of the pandemic. The traffic restrictions imposed as a measure to control of the spread of the virus reduced the EU’s economic activity and generated financial blockages of those companies which were virtually taken into account when forecasting the budgets of the EU Member States.

These three aggravating factors (pandemic, migration under different aspects and overstretching of economic activity) are significant premises for estimating a serious economic crisis, which will affect global society.

In this context, we aim to develop a regional performance model in the sense of the regional administrative capacity’s versatility in relation to these aforementioned phenomena.

The novelty of the present scientific approach lies in identifying and managing a regional leader in terms of socio-economic performance. Moreover, our approach achieves a unified vision of EU regional policy, by standardizing and building on the literature on the subject. Another new element is the emphasis on the need to redefine the EU in performance-related terms.

The paper proceeds as follows. Section 2, Literature Review, discusses relevant models for the topic. It is followed by Section 3, Data and Methodology, in which we highlight the NUTS 2 regional efficiency indicators able to evaluate on a 5-step scale the administrative efficiency data, models and results. This is followed by an analysis of the results in Section 4. An overview in Section 5 and Section 6 discusses the results and concludes the paper.

## 2. Literature Review

Since December 2019, global society has been facing unfavourable conditions regarding economic growth against the background of the COVID-19 pandemic and the necessary global measures to limit the spread of the pandemic.

Although up to this date, the global economy was on an upward slope with normal fluctuations induced by business cycles, the effect of the pandemic downturn has been to freeze the upward slope, as global society is currently facing a significant economic downturn, in the form of short-circuiting the traditional mechanisms of financial, social and security balance of the global market. The factor perpetuating the effects of the pandemic is population migration, which, in this case, contributed to the amplification of the pandemic’s effects, generating the multiplication of COVID-19 outbreaks in multiple global regions, from the initial Wuhan centre to Europe, via Italy and America, where the most disastrous effects of the pandemic are currently manifesting.

Faced with the economic downturn, the states of the world have been confronted with an atypical situation against which the traditional models developed by specialists over time and the efforts of sustainable economic growth are annihilated, both in terms of their effects and the expected economic results. In this context, there is a need for a new approach to address the vulnerabilities of classical theories due to the disregard of the exaggerated values of the pandemic, the social and economic risk of disturbing factors which can act on the global economy at a given time. The effect of this action constitutes the global economic crisis itself.

In this gear, several factors related to the specificities of the global economy, namely transactions in financial markets, the role of single currencies, the mechanism of global financing and the interconnection between national economies through multinational companies, are introduced.

From the regional point of view, there is a direct induced reflection of the global economic crisis on regional autonomy by destabilizing the financial equilibrium levers that highlight the difficulty of the regional territorial administrative units and which weaken the effects of the social protection and the sustainable development programs that they had in the usual course. 

A summary of the previous research on the effects of the disturbing factors on economic growth, as well as the expiration of the previous models through the effect of disturbing factors, is presented in Table 1.

The analysis of the specialized literature on all the major disturbing aspects that are the subject of the present study supports the opportunity to draw up in the current context a new integrated model for analysing the regional performance of the administrative capacity through the indicators with an essential impact on the disturbance.

## 3. Data and Methodology

In order to achieve the major objective of the research, namely the analysis of regional performance according to the versatility of administrative capacity in relation to disturbing factors (the COVID-19 crisis, the induced economic crisis and the migration from Europe manifested in recent years), we proceeded to interrogate the Eurostat database in order to obtain a series of data on NUTS 2 regional efficiency indicators, such as:

I1. Investment share of GDP by institutional sectors, predicted as % of GDP total investment, Eurostat code: 0sdg_08_11
I2. Early leavers from education and training by sex, predicted as % of population aged 18 to 24, Eurostat code: 0sdg_04_10
I3. Gross domestic expenditure on R&D by sector, predicted as % of GDP, Eurostat code: 0sdg_09_10
I4. Employment in high- and medium-high-technology manufacturing and knowledge-intensive services, predicted as % of total employment, Eurostat code: 0sdg_09_20
I5. People at risk of poverty or social exclusion, predicted as percentage, Eurostat code: 0sdg_01_10
I6. People at risk of income poverty after social transfers, predicted as percentage, Eurostat code: 0sdg_01_20
I7. People living in households with very low work intensity, predicted as percentage of total population aged less than 60, Eurostat code: 0sdg_01_40
I8. Share of renewable energy in gross final energy consumption by sector, predicted as % Renewable energy sources, Eurostat code: 0sdg_07_40
I9. Real GDP per capita, predicted as chain-linked volumes (2010), EUR per capita, Eurostat code: 0sdg_08_10 GDP
I10. Long-term unemployment rate by sex, predicted as % of active population, Eurostat code: 0sdg_08_40
I11. R&D personnel by sector, predicted as % of active population, Eurostat code: 0sdg_09_30
I12. Patent applications to the European Patent Office (source: EPO), predicted as number, Eurostat code: 0sdg_09_40
I13. Employment rates of recent graduates by sex, predicted as % of population aged 20 to 34 with at least upper secondary education, Eurostat code: 0sdg_04_50
I14. Energy import dependency by products, predicted as % of imports in total energy consumption, Eurostat code: 0sdg_07_50

These indicators have been selected from specific statistical indicators used by Eurostat in its sustainability analysis (https://ec.europa.eu/eurostat/web/sdi/indicators (accessed on 2 March 2021)). The selection of indicators was based on the relevance of the data to the topic under analysis.

**Figure 1:**
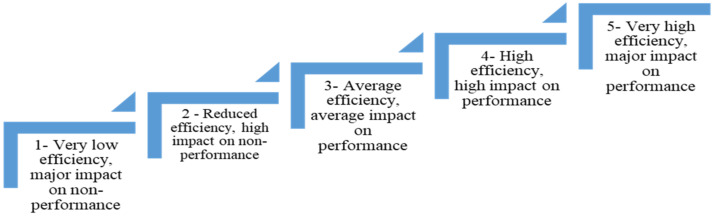
The 5-step scale for administrative efficiency.

For the assessment of the initial regional administrative capacity (before manifesting the disturbing factors potentiated by the COVID-19 epidemic), the authors proceeded to evaluate on a 5-step scale the administrative efficiency. The scale was adjusted to the values reported by Eurostat for the aforementioned indicators and allowed by using the mean, the median and the module to classify each NUTS 2 region for the 14 indicators on one of the 5 steps of the scale, as defined in Figure 1.

**Figure 2:**
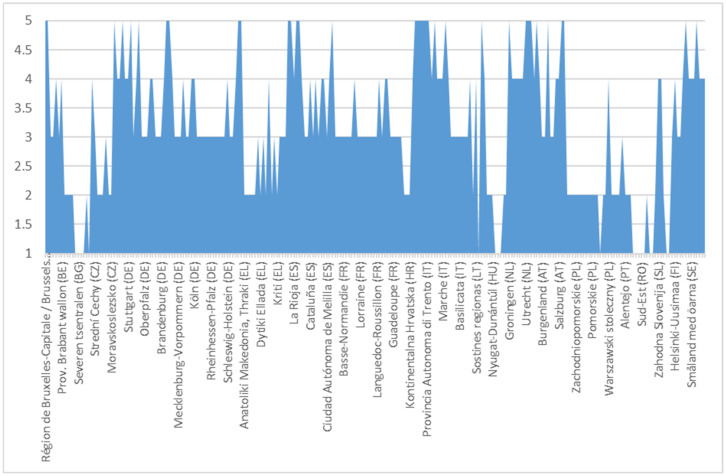
Regional efficiency chart on NUTS 2 regions.

**Figure A1:**
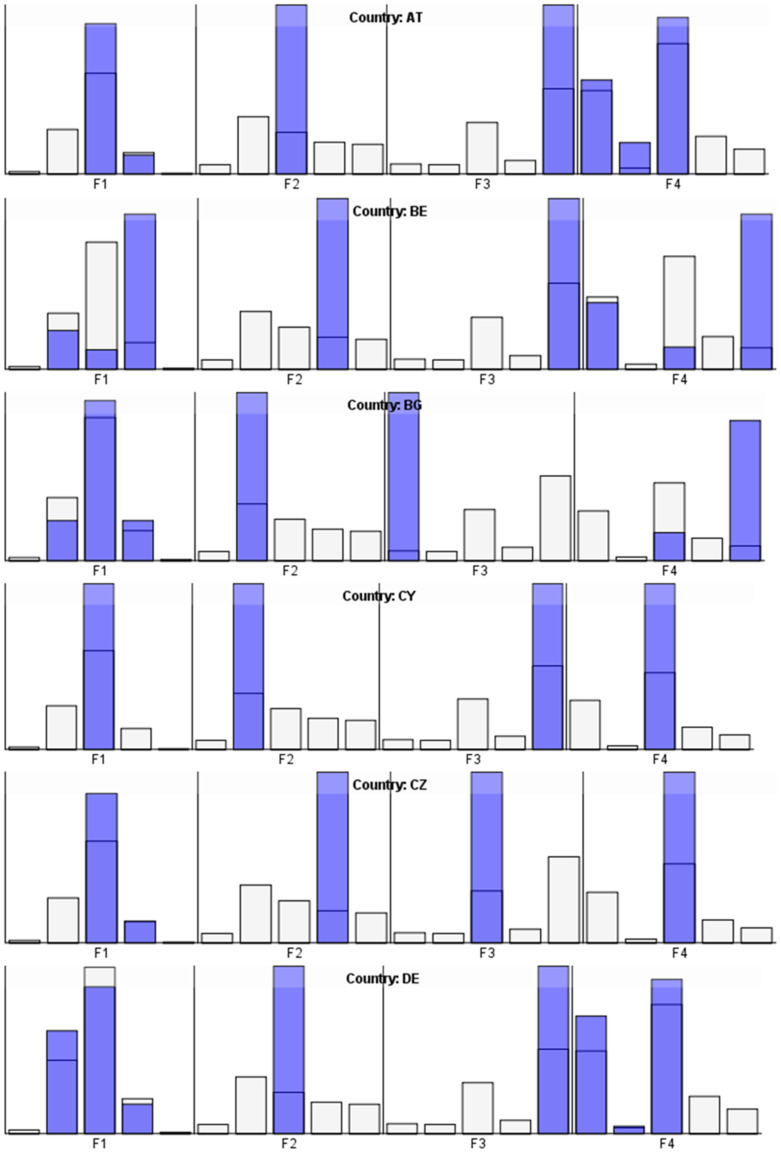

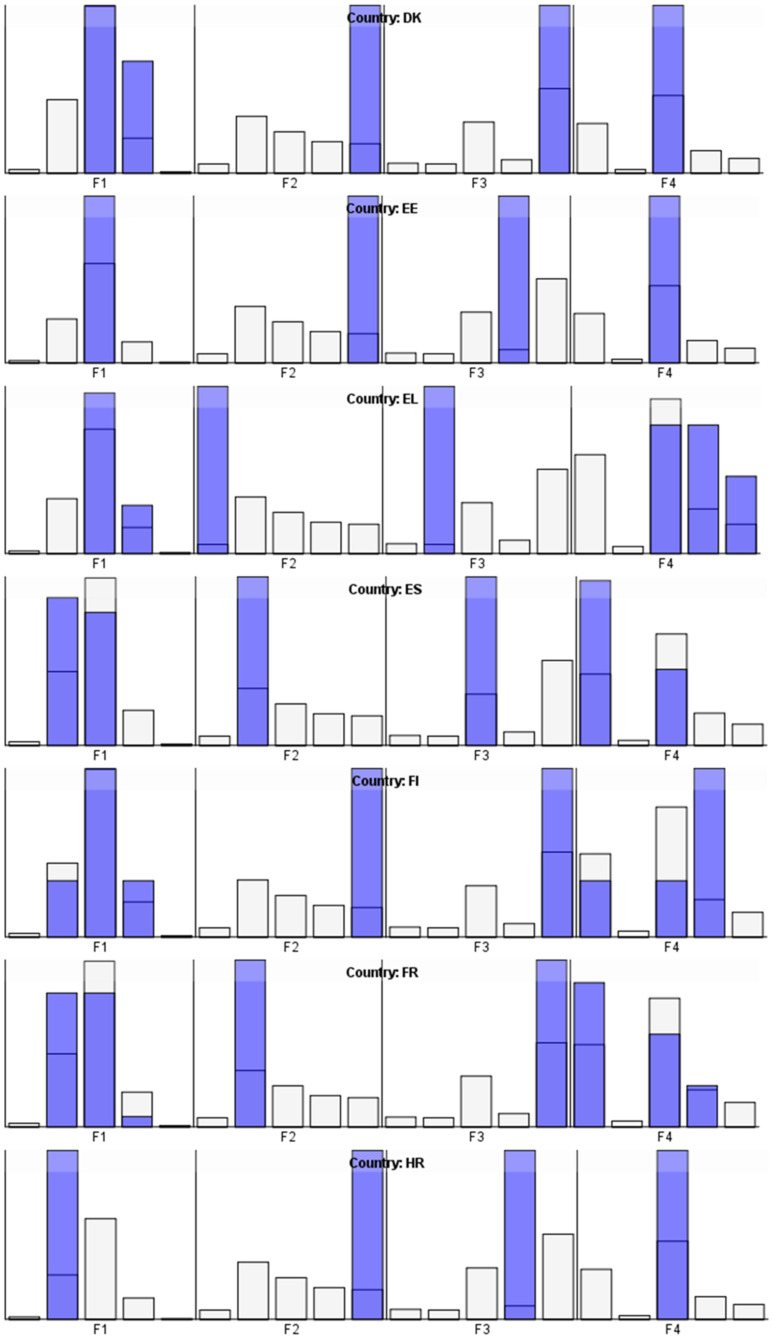

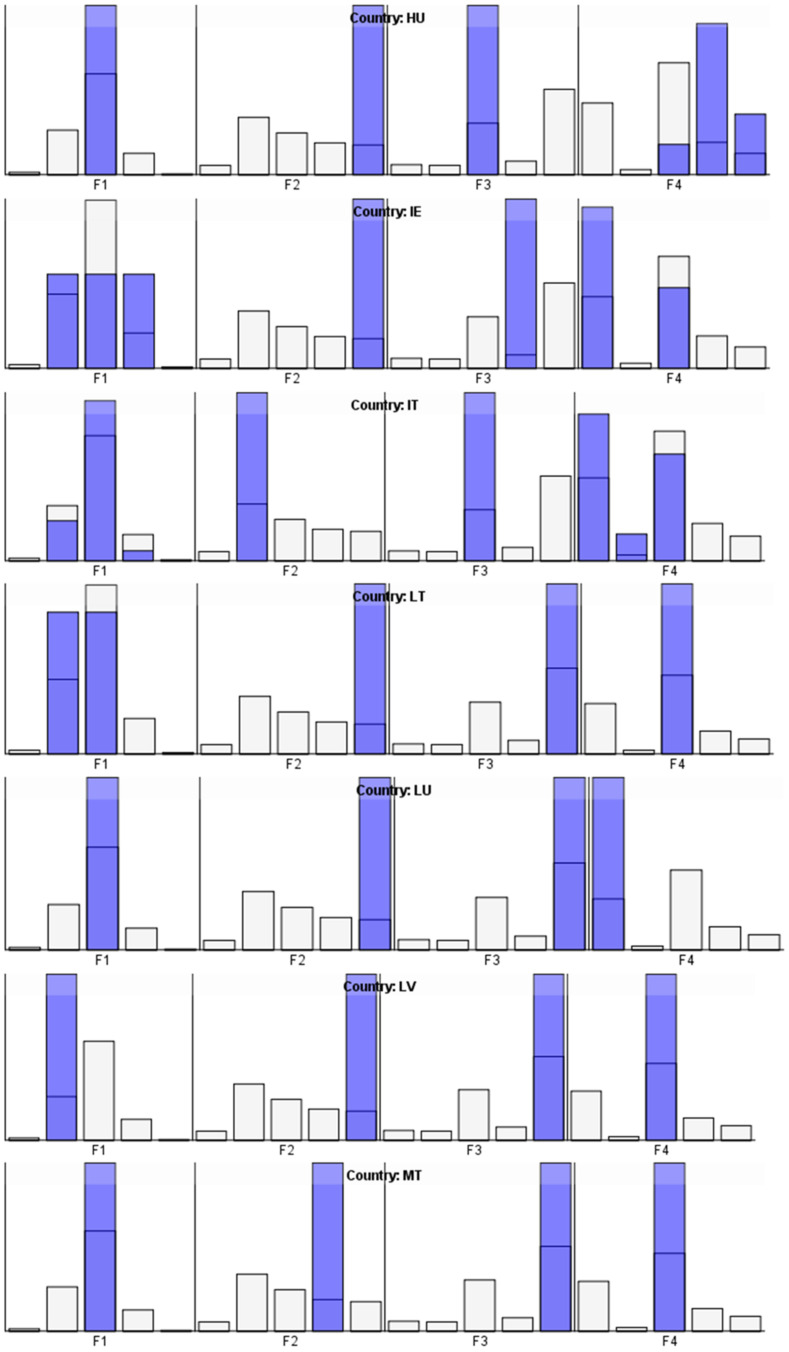

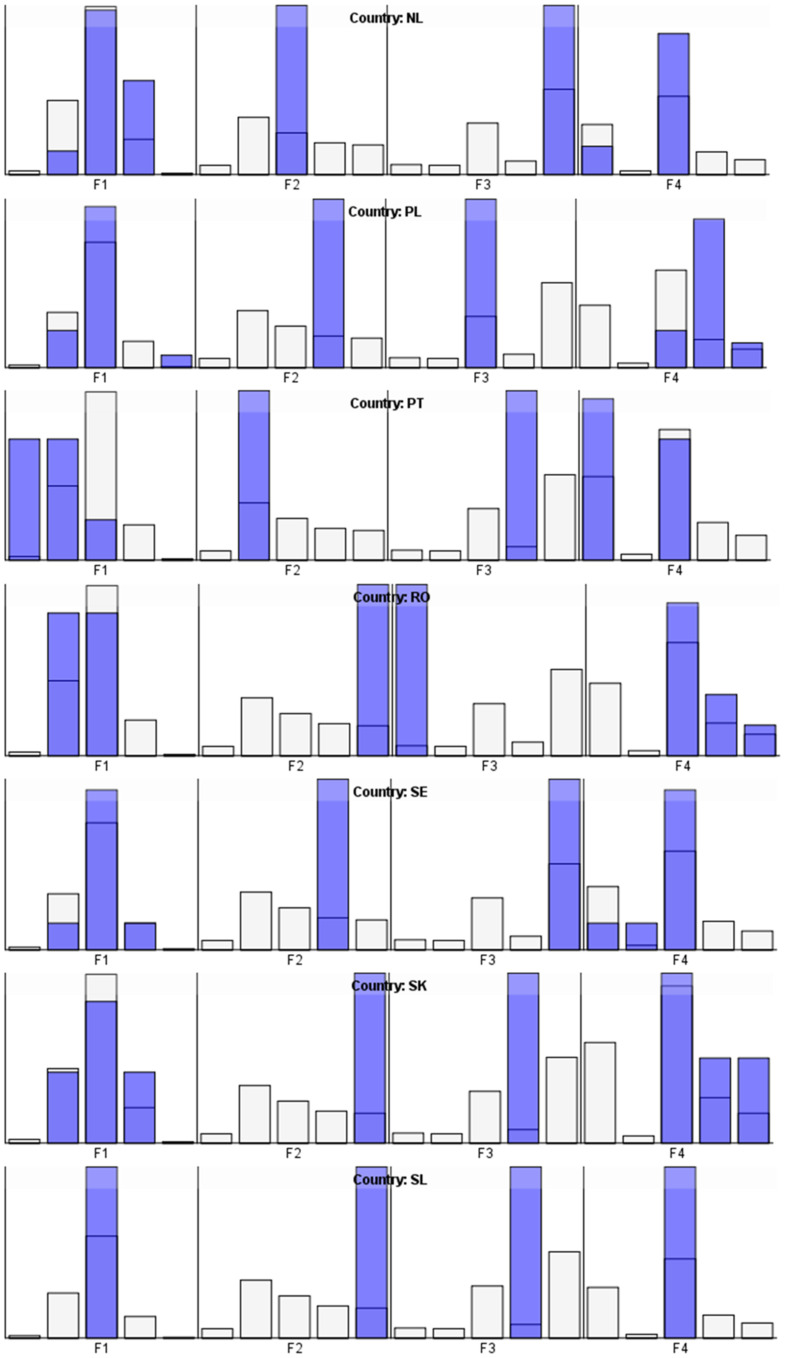
F1—Economic crisis’ impact; F2—Refugees’ impact; F3—Migration’s impact; F4—COVID-19’s impact.

After comparing the data series, a 14-step efficiency chart was obtained for each of the 240 NUTS 2 regions of the EU. This picture, resulting from the application of the individual arithmetic mean to the region, allowed the calculation on the aforementioned scale of basic regional administrative capacity on a performance structure unaffected by the 3 disruptive factors. The graphical centralization of the performance is presented in Figure 2, where due to space restrictions the legend does not show all the regions. These are detailed in the Appendix A (Figure A1).

From Figure 2, it appears that the overall distribution is one in favour of medium efficiency, with a medium impact on performance. There are some situations (8 out of 10 regions) where the scalar level of the reliability indicator is very low, denoting a major impact on non-performance. These regions are located in the east of the EU.

**Figure 3:**
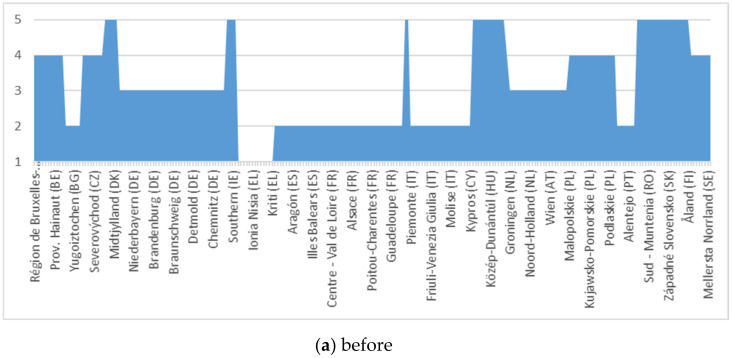

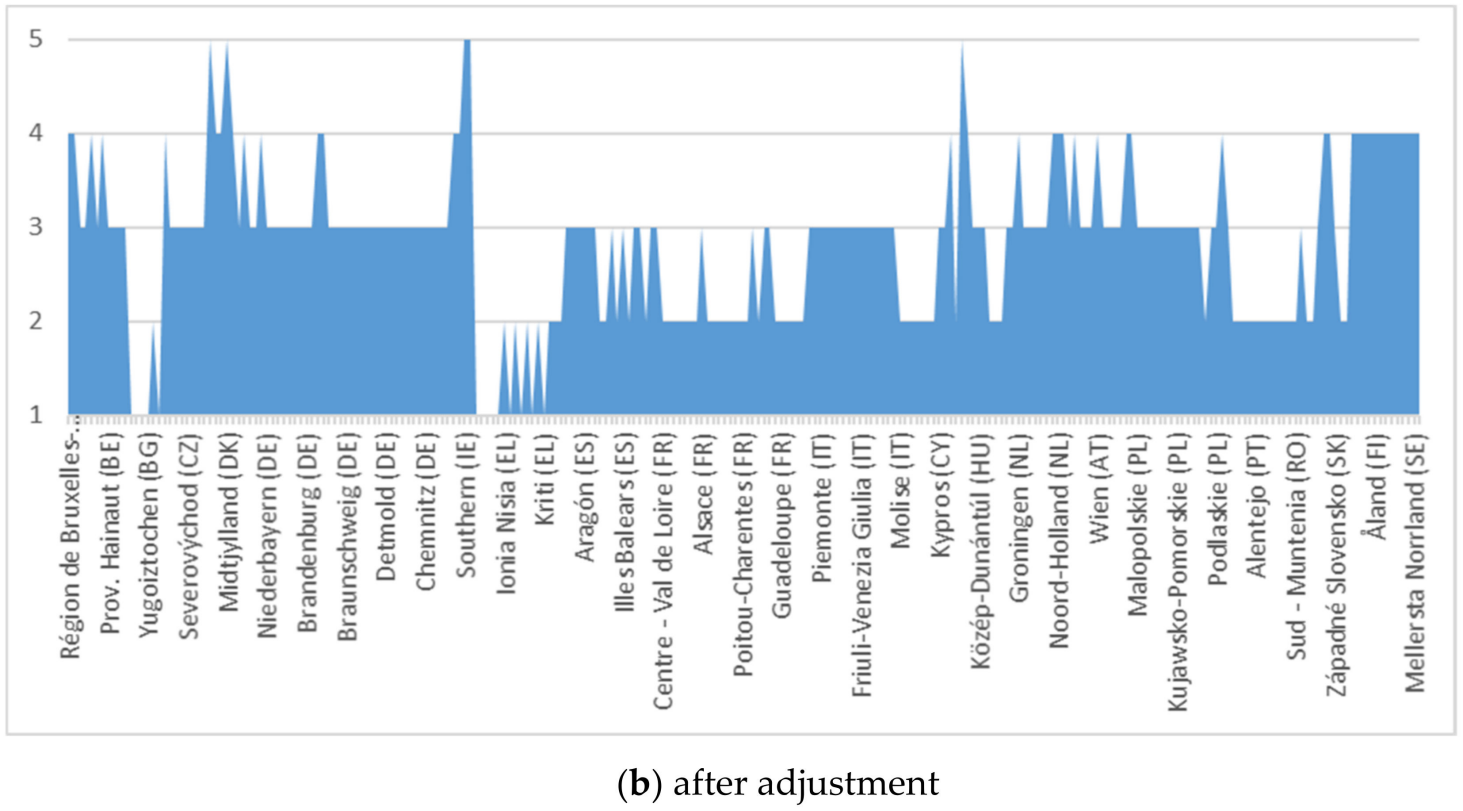
Refugee migration.

The calculation of the coefficients was performed according to the formula:

(1) $$\mathrm{If}\;\underset{t\to \infty }{\lim}\left(\frac{\frac{\left(\sum_{t=1}^{15}I_{i,t}\right)}{\sum_{t=1}^{15}t}}{\frac{\sum_{i=1}^{240}\left(\sum_{t=1}^{15}I_{i,t}\right)}{\sum_{t=1}^{15}t*\sum_{i=1}^{240}i}}\right)\to min\;,\;than\;P_{r_{i,t}}=1$$

(2) $$\mathrm{If}\;\underset{t\to \infty }{\lim}\left(\frac{\frac{\left(\sum_{t=1}^{15}I_{i,t}\right)}{\sum_{t=1}^{15}t}}{\frac{\sum_{i=1}^{240}\left(\sum_{t=1}^{15}I_{i,t}\right)}{\sum_{t=1}^{15}t*\sum_{i=1}^{240}i}}\right)\to 0\;,\;than\;P_{r_{i,t}}=2$$

(3) $$\mathrm{If}\;\underset{t\to \infty }{\lim}\left(\frac{\frac{\left(\sum_{t=1}^{15}I_{i,t}\right)}{\sum_{t=1}^{15}t}}{\frac{\sum_{i=1}^{240}\left(\sum_{t=1}^{15}I_{i,t}\right)}{\sum_{t=1}^{15}t*\sum_{i=1}^{240}i}}\right)=0\;,\;than\;P_{r_{i,t}}=3$$

(4) $$\mathrm{If}\;\underset{t\to \infty }{\lim}\left(\frac{\frac{\left(\sum_{t=1}^{15}I_{i,t}\right)}{\sum_{t=1}^{15}t}}{\frac{\sum_{i=1}^{240}\left(\sum_{t=1}^{15}I_{i,t}\right)}{\sum_{t=1}^{15}t*\sum_{i=1}^{240}i}}\right)\to 1\;,\;than\;P_{r_{i,t}}=4$$

(5) $$\mathrm{If}\;\underset{t\to \infty }{\lim}\left(\frac{\frac{\left(\sum_{t=1}^{15}I_{i,t}\right)}{\sum_{t=1}^{15}t}}{\frac{\sum_{i=1}^{240}\left(\sum_{t=1}^{15}I_{i,t}\right)}{\sum_{t=1}^{15}t*\sum_{i=1}^{240}i}}\right)\to max\;,\;than\;P_{r_{i,t}}=5$$

where: *Pr_i,t_*—regional performance in basic terms, without the effects of the COVID-19 pandemic; *I_i,t_*—the value of the 14 aforementioned indicators for the 240 regions over the 15-year horizon (data collected from Eurostat 2006–2020); *i*—number of EU regions (240); *t*—the time horizon for which the analysis was performed; and coefficients 1, 2, 3, 4, 5—the scalar values shown in Figure 3.

**Figure 4:**
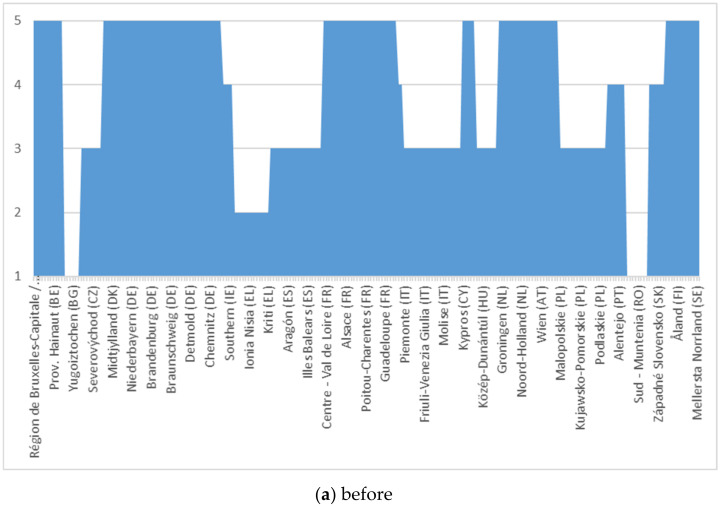

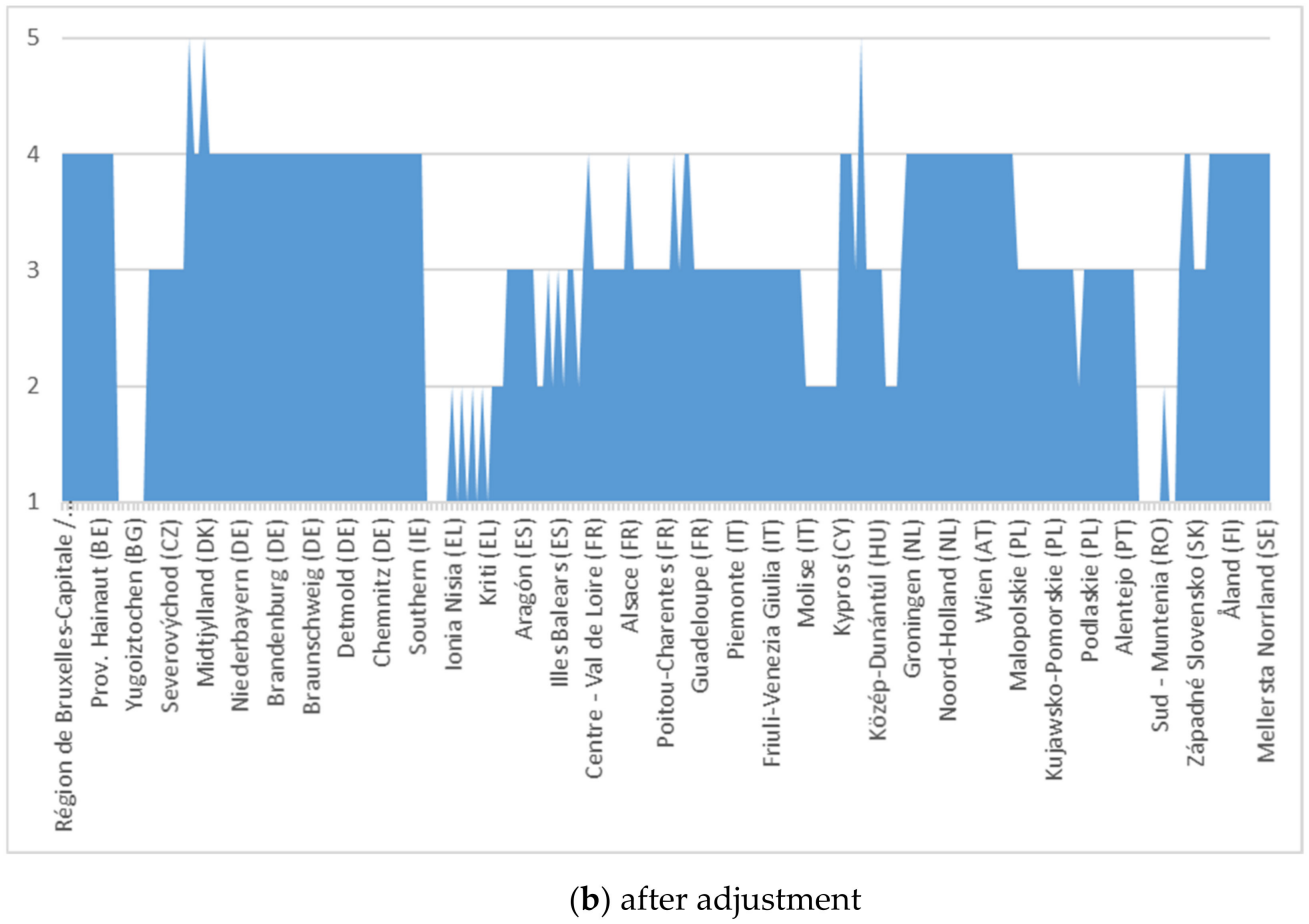
EU population’s migration.

For the scalar evaluation of the European population and of the refugees’ migration phenomenon, we analysed data from the specialized literature and the official figures presented by Eurostat. Finally, we made our own dimension based on the reality in each Member State regarding the migration and its impact on the destination state, as well as on the migration of the European population from states with less developed economies to those with developed economies. The empirically and statistically evaluated data are presented in Figure 3 and Figure 4.

Following the analysis of the migration phenomenon through the lens of the proposed model, there is an increase in the impact of the phenomenon on European regions, especially on those with a low level of influence of the indicators in the initial (unadjusted) version. According to the figure, the trend is one of evenness/reduction in disparities, but towards the high impact area.

In Figure 4, we have analysed the migration of the European population within the Union, showing a more pronounced trend compared to the baseline period, but less pronounced than in the case of refugee migration. Under the impact of the refugee crisis, we estimate that some of the restrictive measures will also have an effect on the migration of the European population, which can be seen by comparing the fitted diagrams in Figure 3 and Figure 4.

**Figure 5:**
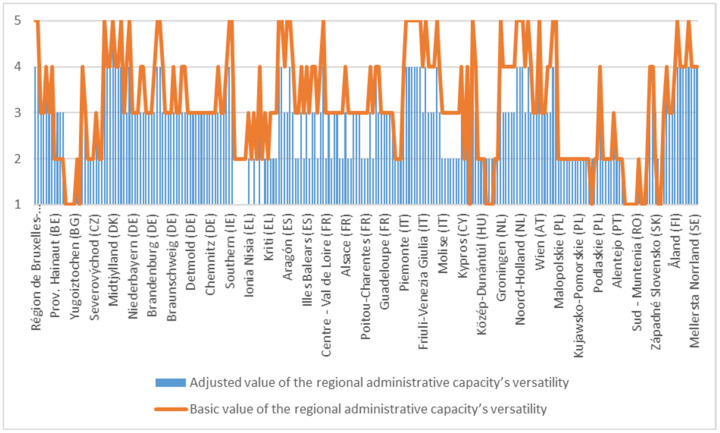
Regional administrative capacity under the conditions of the analysed disturbed factors.

**Figure 6:**
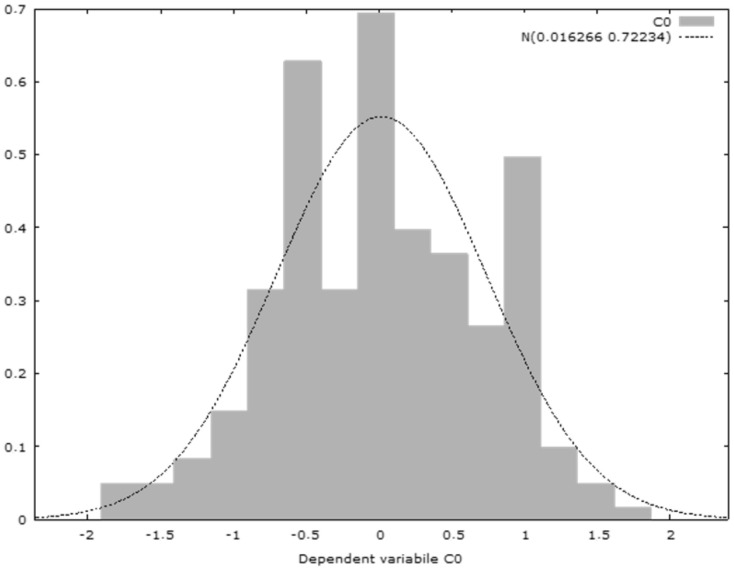
Distribution of the dependent variable.

For the homogeneity of the analysis process, we used the same scale in Figure 3, so that our new model allows data parity. After estimating the data series (point “a” in Figure 5 and Figure 6), their adjustment was made regarding the impact on the versatility of regional administrative capacity through the analysed disturbance factor (internal migration + refugees).

The adjustment model started from the results obtained by calculating the basic administrative capacity at the regional level, on which the consecutive impact of the two phenomena was evaluated according to the formula:

(6) $$\mathrm{If}\;\underset{i\to \infty }{\lim}\left(\frac{M_{i}}{\frac{\sum_{i=1}^{240}\left(M_{i}\right)}{\sum_{i=1}^{240}i}}\right)\to min\;,\;than\;M_{i}=1$$

(7) $$\mathrm{If}\;\underset{i\to \infty }{\lim}\left(\frac{M_{i}}{\frac{\sum_{i=1}^{240}\left(M_{i}\right)}{\sum_{i=1}^{240}i}}\right)\to 0\;,\;than\;M_{i}=2$$

(8) $$\mathrm{If}\;\underset{i\to \infty }{\lim}\left(\frac{M_{i}}{\frac{\sum_{i=1}^{240}\left(M_{i}\right)}{\sum_{i=1}^{240}i}}\right)=0\;,\;than\;M_{i}=3$$

(9) $$\mathrm{If}\;\underset{i\to \infty }{\lim}\left(\frac{M_{i}}{\frac{\sum_{i=1}^{240}\left(M_{i}\right)}{\sum_{i=1}^{240}i}}\right)\to 1\;,\;than\;M_{i}=4$$

(10) $$\mathrm{If}\;\underset{i\to \infty }{\lim}\left(\frac{M_{i}}{\frac{\sum_{i=1}^{240}\left(M_{i}\right)}{\sum_{i=1}^{240}i}}\right)\to max\;,\;than\;M_{i}=5$$

(11) $${P_{r_{i,t}}}^{1}=\sqrt{P_{r_{i,t}}\times M_{i}}$$

where: *M_i_*—the intensity of the migration phenomenon in region *i*, *i* ϵ $\left[1,\;240\right]$, ${P_{r_{i,t}}}^{1}$—adjusting the basic model with the scalar afferent to the migration phenomenon calculated based on the above formula.

(12) $$\mathrm{If}\;\underset{i\to \infty }{\lim}\left(\frac{R_{i}}{\frac{\sum_{i=1}^{240}\left(R_{i}\right)}{\sum_{i=1}^{240}i}}\right)\to min\;,\;than\;R_{i}=1$$

(13) $$\mathrm{If}\;\underset{i\to \infty }{\lim}\left(\frac{R_{i}}{\frac{\sum_{i=1}^{240}\left(R_{i}\right)}{\sum_{i=1}^{240}i}}\right)\to 0\;,\;than\;R_{i}=2$$

(14) $$\mathrm{If}\;\underset{i\to \infty }{\lim}\left(\frac{R_{i}}{\frac{\sum_{i=1}^{240}\left(R_{i}\right)}{\sum_{i=1}^{240}i}}\right)=0\;,\;than\;R_{i}=3$$

(15) $$\mathrm{If}\;\underset{i\to \infty }{\lim}\left(\frac{R_{i}}{\frac{\sum_{i=1}^{240}\left(R_{i}\right)}{\sum_{i=1}^{240}i}}\right)\to 1\;,\;than\;R_{i}=4$$

(16) $$\mathrm{If}\;\underset{i\to \infty }{\lim}\left(\frac{R_{i}}{\frac{\sum_{i=1}^{240}\left(R_{i}\right)}{\sum_{i=1}^{240}i}}\right)\to max\;,\;than\;R_{i}=5$$

(17) $${P_{r_{i,t}}}^{2}=\sqrt{P_{r_{i,t}}\times R_{i}}$$

where: *R_i_*—the intensity of the refugee migration phenomenon in region *i*, *i* ϵ $\left[1,\;240\right]$, ${P_{r_{i,t}}}^{2}$—adjusting the basic model with the scalar of the refugee migration phenomenon calculated based on the above formula.

Regarding the impairment of regional administrative capacity with the scalar values of the impact due to the COVID-19 pandemic in EU regions, we used the data communicated on the statista.com website, which presents in detail the distribution of the regional spread of the diseases with COVID-19 in each Member State. These data were centralized by the authors, compared with the demographic values reported by Eurostat through the population on 1 January by NUTS 2 region persons indicator at the level of 2019, calculating the regional disease rate defined as COVID-19’s rate, according to Appendix B (Table A1).

The scaling procedure was similar to those presented above, namely:

(18) $$\mathrm{If}\;\underset{i\to \infty }{\lim}\left(\frac{COVID-19_{i}}{\frac{\sum_{i=1}^{240}\left(COVID-19_{i}\right)}{\sum_{i=1}^{240}i}}\right)\to min\;,\;than\;COVID-19_{i}=1$$

(19) $$\mathrm{If}\;\underset{i\to \infty }{\lim}\left(\frac{COVID-19_{i}}{\frac{\sum_{i=1}^{240}\left(COVID-19_{i}\right)}{\sum_{i=1}^{240}i}}\right)\to 0\;,\;than\;COVID-19_{i}=2$$

(20) $$\mathrm{If}\;\underset{i\to \infty }{\lim}\left(\frac{COVID-19_{i}}{\frac{\sum_{i=1}^{240}\left(COVID-19_{i}\right)}{\sum_{i=1}^{240}i}}\right)=0\;,\;than\;COVID-19_{i}=3$$

(21) $$\mathrm{If}\;\underset{i\to \infty }{\lim}\left(\frac{COVID-19_{i}}{\frac{\sum_{i=1}^{240}\left(COVID-19_{i}\right)}{\sum_{i=1}^{240}i}}\right)\to 1\;,\;than\;COVID-19_{i}=4$$

(22) $$\mathrm{If}\;\underset{i\to \infty }{\lim}\left(\frac{COVID-19_{i}}{\frac{\sum_{i=1}^{240}\left(COVID-19_{i}\right)}{\sum_{i=1}^{240}i}}\right)\to max\;,\;than\;COVID-19_{i}=5$$

(23) $${P_{r_{i,t}}}^{3}=\sqrt{P_{r_{i,t}}\times COVID-19_{i}}$$

where: *COVID-19*—the intensity of the population infection phenomenon following the outbreak of the pandemic in region *i*, *i* ϵ $\left[1,\;240\right]$, ${P_{r_{i,t}}}^{3}$—adjusting the basic model with the scalar afferent to the phenomenon of population infection following the outbreak of the pandemic, calculated based on the above formula.

In order to evaluate the impact of the economic crisis, a two-step formula was applied. In the first step, the scalar indicators I7, I9 and I13 were used: I7, people living in households with very low work intensity, predicted as the percentage of the total population aged less than 60, Eurostat code: 0sdg_01_40; I9, real GDP per capita, predicted as chain-linked volumes (2010), EUR per capita, Eurostat code: 0sdg_08_10 GDP; and I13, employment rates of the recent graduates by sex, predicted as % of population aged 20 to 34 with at least upper secondary education, Eurostat code: 0sdg_04_50.

In the second stage, the scalar indicator calculated for COVID-19 was used, according to the formula:

(24) $$\mathrm{If}\;\underset{t\to \infty }{\lim}\left(\frac{\frac{\left(\sum_{n=1}^{3}I_{i,n}\right)}{\sum_{n=1}^{3}n}+COVID-19_{i}}{2}\right)\to min\;,\;than\;\;C_{i}=1$$

(25) $$\mathrm{If}\;\underset{t\to \infty }{\lim}\left(\frac{\frac{\left(\sum_{n=1}^{3}I_{i,n}\right)}{\sum_{n=1}^{3}n}+COVID-19_{i}}{2}\right)\to 0\;,\;than\;\;C_{i}=2$$

(26) $$\mathrm{If}\;\underset{t\to \infty }{\lim}\left(\frac{\frac{\left(\sum_{n=1}^{3}I_{i,n}\right)}{\sum_{n=1}^{3}n}+COVID-19_{i}}{2}\right)=0\;,\;than\;\;C_{i}=3$$

(27) $$\mathrm{If}\;\underset{t\to \infty }{\lim}\left(\frac{\frac{\left(\sum_{n=1}^{3}I_{i,n}\right)}{\sum_{n=1}^{3}n}+COVID-19_{i}}{2}\right)\to 1\;,\;than\;\;C_{i}=4$$

(28) $$\mathrm{If}\;\underset{t\to \infty }{\lim}\left(\frac{\frac{\left(\sum_{n=1}^{3}I_{i,n}\right)}{\sum_{n=1}^{3}n}+COVID-19_{i}}{2}\right)\to max\;,\;than\;C_{i}=5$$

(29) $${P_{r_{i,t}}}^{3}=\sqrt{P_{r_{i,t}}\times C_{i}}$$

where: *C_i_*—the scalar coefficient of the economic crisis impact on the regional administrative capacity calculated according to the above formula; $I_{i,n}$—scalar indicators I7, I9 and I13; ${P_{r_{i,t}}}^{4}$—adjusting the basic model with the scalar afferent to the phenomenon after the outbreak of the pandemic, calculated based on the above formula.

The composite value of the versatility indicator of regional administrative capacity in relation to the 3 disturbing phenomena (economic crisis, COVID-19 pandemic and migration) was calculated based on the 4 intermediate values, adjusted by rounded arithmetic mean to the value of the scalars defined in Figure 1, according to the formula:

(30) $${P_{r_{i,t}}}^{*}=\frac{{P_{r_{i,t}}}^{1}+{P_{r_{i,t}}}^{2}+{P_{r_{i,t}}}^{3}+{P_{r_{i,t}}}^{4}}{4}$$

The graphical representation of the predicted final values for the analysed indicator, namely the versatility of the regional administrative capacity under the conditions of the analysed disturbed factors, is shown in Figure 5:

From Figure 5, it emerges that the final predictive values for regional administrative capacity versatility under the disturbing factors denote an evolving amplitude for most regions. There are cases where the versatility of regional administrative capacity is reduced by at least one point of influence from the base level. This means that disturbing factors constitute monitoring elements for the regional administrations on the basis of which the regional policies can be rebuilt in the short and medium term, with the advantage of maintaining the stability of the regional capacity by mitigating or eliminating the influence of these disturbing factors.

## 4. Results

We designed an econometric model in order to test the correlation between the previously presented variables, with the title of global phenomena with negative impact, namely the economic crisis, the refugees and the migration, and not lastly, the impact of the COVID-19 pandemic. These variables allowed the calculation of the scalar coefficients of regional administrative capacity in the absence of the disturbing factors (C0) and in the presence of each individual (C1–C4) in order to finally allow the calculation of the regional administrative capacity versatility under the current crisis conditions. The definitions of the aforementioned terms are:

F1—Economic crisis’ impact;
F2—Refugees’ impact;
F3—Migration’s impact;
F4—COVID-19’s impact;
C0—Basic regional administrative capacity;
C1—Refugees’ impact on regional administrative capacity;
C2—Migration’s impact on regional administrative capacity;
C3—COVID-19’s impact on regional administrative capacity;
C4—Economic crisis’ impact on regional administrative capacity;
R—Regional administrative capacity.

The new proposed model is based on the following hypotheses H1–H3:

**Hypothesis** **1** **(H1).** *There is a direct and quantifiable dependence between the regional administrative capacity and the regional economic power quantified in real GDP/capita. In the absence of the disturbing factors, it is estimated that the growth trend of the regional economies is directly related to the regional administrative capacity and in terms of economic growth in the EU as a whole.*

**Hypothesis** **2** **(H2).** *In the presence of the disturbing factors, it is estimated that the greatest impact quantified by the correlation between the scalar of the phenomenon and the impact on the regional administrative capacity is generated by F4 (COVID-19 pandemic) and F1 (Economic crisis’ impact).*

**Hypothesis** **3** **(H3).** *There is a demonstrable econometric correlation through the same dependent variable between the scalar value of the disturbing factors with regional manifestation and the scalar value of the regional administrative capacity through a high statistical confidence regarding the test of bivalent correlation between C0, R and the factors Fi.*

In the above context of the hypotheses and the methodological concepts presented in Section 3, we define the correlational econometric model based on the two-stage least squares regression. The model used Gretl software version 2019, according to the regression equation as follows:

(31) $$\left\{\begin{array}{c} C0=+2.19\times F1+0.105\times F2+0.0501\times F3-1.34\times F4\\ \langle n\;=240.\;R-\mathrm{squared}\;=0.729\rangle \\ C0=+1.13\times R\\ \langle n\;=240.\;R-\mathrm{squared}\;=0.968\;\rangle \end{array}\right.$$

According to this regression equation, we find highly significant statistical values of the model for the dependent variable C0, the regressors Fi and the instrumented variables Ci. In this case, the statistical significance test indicates the value of 72.9%, while the C0-R correlation indicates a 96.8% value of the statistical significance test, which leads to a first conclusion of the model’s validity. In order to consolidate this conclusion, the statistical tests were performed for the TSLS model applied to the EU 240 NUTS 2 regions, obtaining significant values of the *p*-value indicator for the variable regression scales F1, F4 and F2 (see Table 2). The *p*-value is medium significant for F2, while it is highly significant for F1 and F4.

The heteroscedasticity test reveals that, in the case of the null hypothesis, the heteroscedascity is not present, and the test for the residual normality reflects that the error is normally distributed, according to the distribution diagram of the Gaussian curve.

Hausman test—null hypothesis: OLS estimates are consistent; asymptotic statistical test: Hi square (4) = 728.81 with *p*-value = 2.01207e^−^^156^; Pesaran–Taylor test for heteroskedasticity—null hypothesis: heteroscedasticity is not present; asymptotic statistical test: z = 0.329671 with *p*-value = 0.741648; test for residual normality—null hypothesis: the error is normally distributed; statistical test: Hi square (2) = 2.79135 with *p*-value = 0.247665; weak instrument test—Cragg–Donald minimum eigenvalue = 50.9864 (see Figure 6).

**Figure 7:**
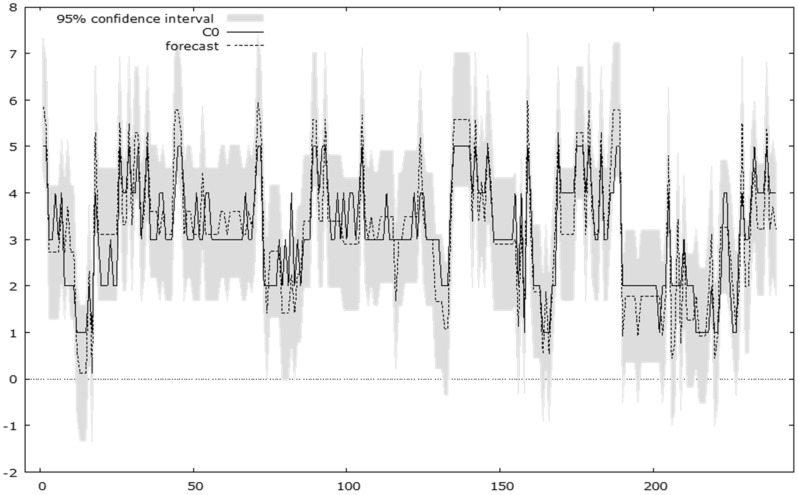
Predicted distribution diagram of the dependent variable for the 95% confidence interval.

The predicted analysis of the amplitude of the dependent variable variation achieves over the 95% confidence interval and shows a significant impairment of the values of the basic regional capacity (C0) in the presence of the disturbing factors. This forecast is shown in the diagram (Figure 7):

The model proposed in this paper objectively evaluated the versatility of the regional administrative capacity in relation to the disturbing factors, finding a high influence of F1 and F4 and an average influence of F2 on the dependent variable. Variable F3 represents a residual regressor in the proposed model because it is concerned with the measures adopted during the pandemic and economic crisis to limit the movement of people and to control the social distance.

## 5. Discussion

Through the analysis performed during the present research, the working hypotheses were demonstrated as follows:

**H1** **demonstration:** *There is a direct and quantifiable dependence between the regional administrative capacity and the regional economic power quantified in real GDP/capita, so that, in the absence of disturbing factors, it is estimated that the growth trend of the regional economies is directly related to the regional administrative capacity and in terms of economic growth in the EU as a whole. This aspect was also highlighted by Asahi K, Undurraga EA, Valdés R and Wagner R. (2021) [15], who pointed out that, during the crisis period, there is an impairment of the regional economy compared to the development experienced by these regions before the crisis (see Table 1).*

This aspect was demonstrated because for the calculation of the regional administrative capacity, 14 regional statistical indicators were integrated: I1. investment share of GDP by institutional sectors, I2. early leavers from education and training by sex, I3. gross domestic expenditure on R&D by sector, predicted as % of GDP, I4. employment in high- and medium-high-technology manufacturing and knowledge-intensive services, I5. people at risk of poverty or social exclusion, I6. people at risk of income poverty after social transfers, I7. people living in households with very low work intensity, I8. share of renewable energy in gross final energy consumption by sector, I9. real GDP per capita, I10. long-term unemployment rate by sex, I11. R&D personnel by sector, I12. patent applications to the European Patent Office, I13. employment rates of recent graduates by sex and I14. energy import dependency by products.

(32) ^ I9. real GDP per capita=+0.936×C0. basic regional administrative capacity

*n* = 240, R-squared = 0.956.

**Figure 8:**
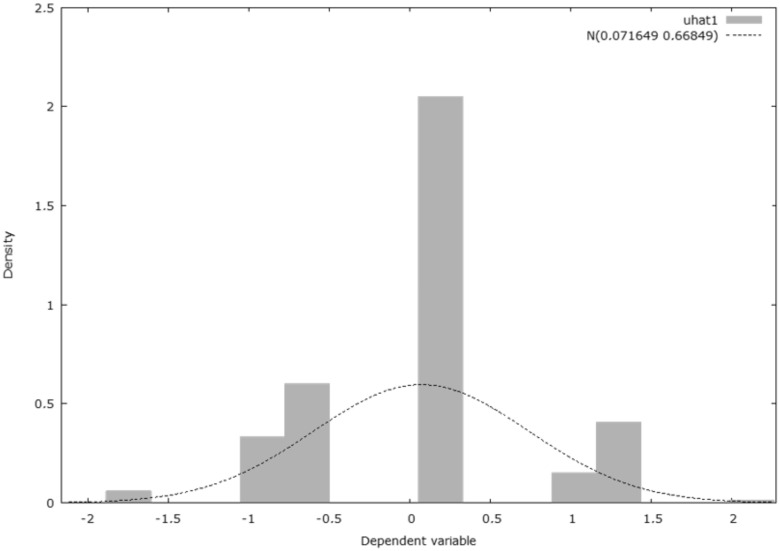
The correlation diagram.

The correlation diagram is shown in Figure 8.

**H2** **demonstration:** *In the presence of disturbing factors, it is estimated that the greatest impact quantified by the correlation between the phenomenon scalar and the impact on the regional administrative capacity is generated by F4 (COVID-19 pandemic) and F1 (Economic crisis’ impact). In the paper “Economic interventions to ameliorate the impact of COVID-19 on the economy and health: an international comparison,” [13] the authors show that the immediate measures taken by the authorities in the developed countries have a high fiscal impact in terms of combating the effects of the pandemic. Moreover, the authors show that there are common elements in the strategies adopted, namely reducing taxes and adopting measures to support the population. (see Table 1).*

As we demonstrated in Section 4, the *p*-values of the two scalar factors are less than 0.0001 and are smaller than the other 2 scalar factors (F2, F3), contributing to the homogeneity of the proposed model.

**H3** **demonstration:** *There is a demonstrable econometric correlation through the same dependent variable between the scalar value of the disturbing factors with regional manifestation and the scalar value of the regional administrative capacity through a high statistical confidence regarding the test of bivalent correlation between C0, R and the factors Fi. Some authors [6] show that the effect of population health on economic growth can be assessed by a directional model of valuation which, we believe, would be adjusted by the impact value of the disturbing factors. Additionally, other authors [7] consider that the impact of the pandemic on the health status of the population can be measured directly and on the pandemic indirectly based on a Markov chain model, demonstrating the need for a review of public health policies and measures to be implemented to reduce uncertainty. Some researchers [8] presented an analysis of the impact of the disruptive factors on the global economy during the economic crises. They show that these factors act as markers of the flattening of the growth curve. As shown in Table 1, their proposed model can be improved by adding social protection variables with an impact on the economic recovery.*

The correlation is demonstrated for a high level of statistical representativeness, 96.8% for the C0-R correlation and 72.9% for the C0-Fi correlation. These values were presented together with the model equation in Section 4, demonstrating the correlations presented in the Methodology and indicating, together with the statistical tests, that the proposed model is reasonable, homogeneous, well determined and representative in relation to the analysed disturbing phenomena. 

The objectives of the study are aimed primarily at the structured assessment of regional administrative capacity in the initial version, based on statistical indicators, and in the current version, after the outbreak of the pandemic, based on quantifying the impact of the disturbing factors.

**Figure 9:**
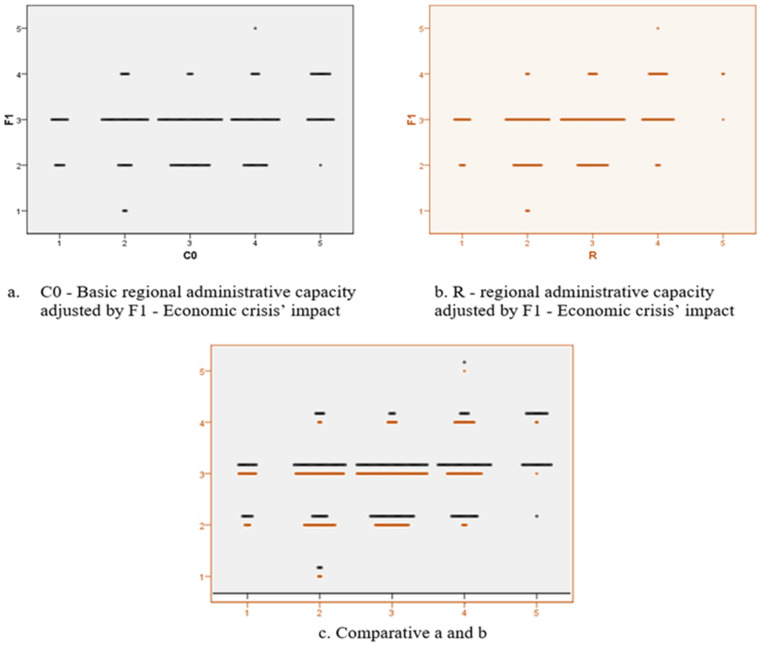
Two-dimensional plot diagram for analysing the C0-R variables in relation to F_1_.

The first objective defined according to the above was achieved by the authors carrying out a structured analysis of regional administrative capacity, based on the disturbing factors, obtaining the following results:

The regional administrative capacity is affected for the F1 scale in the critical value range 1–2, on the segment of the regional economies whose basic variable of the administrative capacity registers values towards the minimum interval (the lower values of the pre-crisis administrative capacity, assessed on the basis of the 14 Eurostat statistical indicators, lead to a higher impact of the economic crisis that disturbs the administrative capacity in the period after this phenomenon occurs). In the same sense, impairments are also found for the strengthened administrative capacities, in the sense of reducing or declassifying those to the average area of the range of scalar values (see Figure 9).

**Figure 10:**
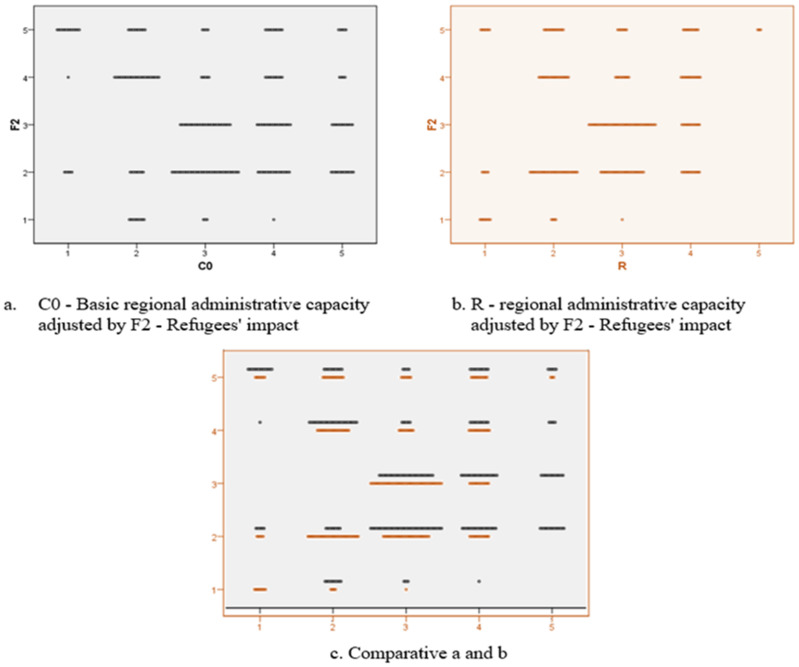
Two-dimensional plot diagram for analysing the C0-R variables in relation to F_2_.

In the case of factor F2 (the impact of refugees), there is a major impairment of administrative capacity for those regional units whose basic administrative capacity C0 registers scalar values in the critical interval (1–2). It can be seen from the figure below (Figure 10) that the administrative capacities that had higher positions in the scalar ranking preceded by the crisis will not be significantly affected by the phenomenon, except the declassification from the maximum category to category.

**Figure 11:**
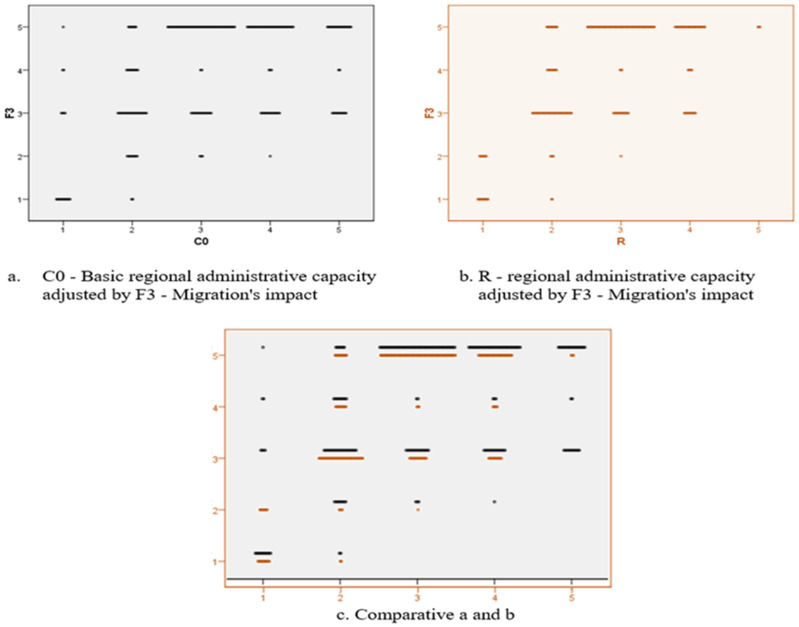
Two-dimensional plot diagram for analysing the C0-R variables in relation to F_3_.

A situation similar to the F2 phenomenon, but with a high impact in the area of the average declassification of the regional administrative capacities, is the phenomenon of the migration of European citizens inside the community space. On the upper segment, the analysis does not involve significant disturbances (see Figure 11).

**Figure 12:**
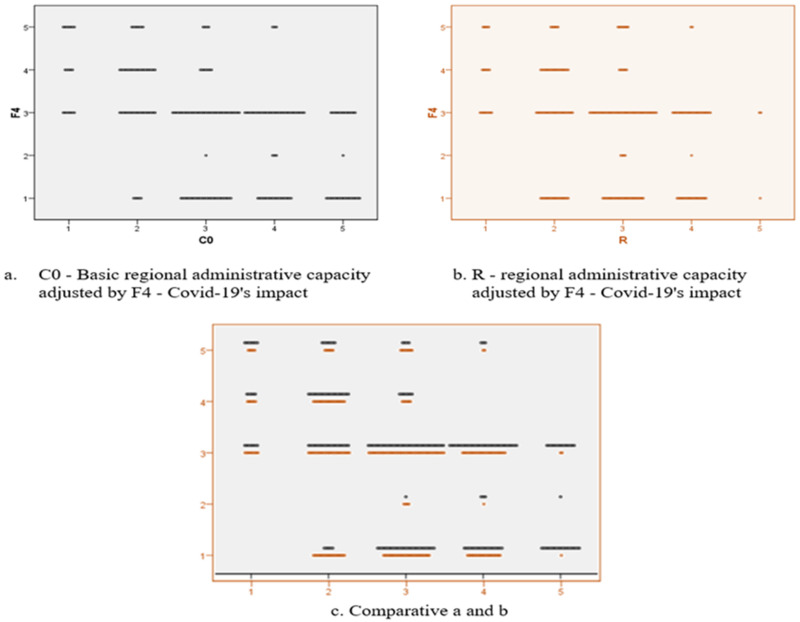
Two-dimensional plot diagram for analysing the C0-R variables in relation to F_4_.

The F4 and F1 factors are the real challenges for the current European context. In the case of F4, there is an increase in the vulnerabilities of the regional administrative capacity both for the regions in the upper echelon and for those in administrative difficulty, because the COVID-19 pandemic socially affects the regions in which it is triggered and requires economic support measures for liquidation of its propagation. According to Figure 12, a translation of the adjusted scalar value F4 of the administrative capacity against the background of the pandemic in the upper echelon is observed, and a maximization of the reduced values for the administrative capacities already in difficulty, according to C0.

The second objective of the study aims to evaluate the reaction of the administrative units according to their capacity in the economic problems in the region, in the sense of improving the performance of the regional economies.

The aspects discussed in the case of the F1 impact (economic crisis) are likely to recommend for the crisis period the implementation of specific measures to increase the regional administrative capacity through proactive methods of supporting the economy, including the improvement of the administrative apparatus relationship with the business environment, creating some databases for connecting small producers with communities that can generate demand for goods and services, setting up e-commerce platforms managed by economic levers to help SMEs, creating a mechanism of decision-making transparency through online portals to help the economic entities affected by the crisis and the improvement and intensification of the activity of the unemployment offices in order to redistribute the surplus of labour on the market affected by the crisis.

Following the regression of correlation analysis of the administrative capacity variable in relation to the impact factor F2, the need to adopt at the EU level some measures to help the affected regions by primary migration was identified, in order to provide technical and financial assistance for weighting the migration phenomenon in the affected areas and to provide superior management of the situation. In this regard, some projects of social insertion and the elimination of any kind of discrimination can be primarily directed to the areas identified as having difficulties, these areas being represented graphically and in the Figure A1 in the Appendix A.

From the analysis of the community migration impact, we found that there is an impairment of the administrative capacity of the reduced impact model (as shown by the model presented in Section 4), on which it was determined that F3 has a low impact in relation to the other factors (*p*-value having the highest coefficient according to the statistical tests performed by modelling). In practice, there is a latent labour demand in the developed regional economies for the primary and secondary sectors; however, community migration is not represented only by the active labour, and there is a population that, due to conjugate factors, fails to reach the developed region and to integrate into the labour market, becoming a social burden for the new region. This population segment represents a real challenge for the regional economies, which will have to identify, in times of economic crisis, real possibilities of engaging them in economic activity, based on the fact that this segment of population that is uninsured and unable to contribute to the regional economy will be at the forefront of potential social conflicts.

The COVID-19 pandemic represents an unprecedented global challenge that has led to a major rate of disease and death among the population, especially the aging population. The cumulative administrative burden cannot be managed in this regional context, with many Member States adopting national strategies and declaring a state of national emergency to prevent the spread of the disease.

The first major effect of the pandemic is the short-circuiting of the health system, given that it was not designed to cope with such a pandemic. At the same time, a food and disinfectant crisis can be induced successively, which raises challenges for the big regional units in order to ensure the optimal conditions of hygiene and health for the regional population in public areas, public transport and in spaces destined for commercial activities.

Some of the regions with low C0 values (the NE region of Romania, for example) encountered, at the outbreak of the pandemic, major problems in the management of the three disruptive factors, which required the institutionalized intervention of the armed forces, and of the ministry of internal affairs to manage the situation, which had escaped control in the region and was resolved with a far greater number of victims than the national average.

## 6. Conclusions

The study carried out in this paper constitutes, by the amplitude of the analysed phenomenon and by the proposed model, a step forward for forecasting the need to strengthen the regional administrative capacity in European space, and it is intended to be a useful tool for competent bodies in the management of the territorial level.

The authors intend to use the results of this research to conduct a further regional impact study, which offers concrete solutions to regional decision makers, in order to efficiently manage the current crisis and its consequences in the short and medium term.

Following the study, it was found that it is necessary to implement immediate economic recovery measures according to the ones presented in the Discussion section, measures that should be integrated into regional plans with economic–administrative specificity.

The results of the present study can be implemented in order to increase the effectiveness of public policies at the regional and local level in the fight against the negative impact of the pandemic on society.

From our point of view, the local and regional public administrations should establish best-practice models in the fight against the effects of COVID-19, and should increase administrative capacity. A starting point in this approach can be the monitoring of the indicators proposed in this paper, and the adoption of an adaptive policy such as the one we have formulated.

The limitations of the study include the introduction of a restricted number of disturbing factors, but the proposed model allows us to remedy this disadvantage by introducing other variables into the analysis.

## Figures and Tables

**Table 1:** Literature review.

No	Authors	Model’s Characteristics	Criticism	Proposals/Solutions
1.	Iamsiraroj, S., 2016 [3]	The author analyses the connection between FDI and economic growth based on extensive statistics for a number of 124 states during 1971–2010, according to the following scheme:	The model proposed by the author could not anticipate the effect of the evolution of the disturbing factors on economic growth. As a result, the value of the β coefficients is estimated as positive while, in our opinion, the correct approach for β is $\beta_{<}^{\geq }$0.	Economic growth can be approached from an inter-disciplinary perspective, provided the β coefficients are correctly estimated. In our study, the economic growth is approached both at the regional level (as a factor of the coefficients’ diversification) and in terms of connections with other macro indicators (migration, population at risk of poverty, etc.).
2.	Meyer, D. and Shera, A., 2017 [4]	The authors have developed an econometric model regarding the effect of the disturbing factors on economic growth. These factors are attributed to the FDI decrease, migration from less developed countries to developed countries and the transactions’ cost due to technological progress. There is an impact on the economic growth at the level of the GDP due to the value of the exchange rate, the debt increase and the aging of the population.	The model benefits from an unjustified optimism regarding the calculation of the positive impact of the increase in schooling rate and of household consumption. A rigid curve influenced in the sense of flattening the regional development’s differences is reached in the sense of less significant factors than those affecting today’s global economic development.	We believe that economic growth based mainly on consumption is not able to ensure a balanced and sustainable development of the economy. For this reason, our proposed model is not predominantly based on consumption demand, but on sustainable development objectives able to make the economic growth curve more flexible and sustainable, with beneficial effects on the whole economic system.
3.	Pradhan, R.P., Arvin, M.B., Hall J.H. and Nair M., 2016 [5]	The authors used a self-regressive vector to highlight the interdependencies between the financial innovation and the economic development in 18 Euro area countries during 1961–2013. The model based on the scenario method (5 scenario) takes into account a significant growth of the economy during the analysed period in net value at a significance rate of 5% in all scenarios, in the context of the manifestation of the limited growth conditions of the patents/inhabitant and of the financial composite index of the development.	This model indicates that the long-term economic growth is stable, but does not highlight the disruptive effect of situations such as the pandemic and economic crisis.	We consider that this model must be adjusted to more eloquently reproduce the influence of the autoregressive vectors presented during the model.
4.	Bloom, D.E., Canning, D., Kotschy, R., Prettner, K. and Schunemann, J.J., 2019 [6]	The authors developed a directional model for evaluating the effects of population health on economic growth. There is a direct quantifiable impact of population health on economic state based on a classical production function, transformed by the authors.	Some variables such as the effort to maintain the population’s proper state of health during the pandemic and the efforts to prevent and update medical systems slow down economic growth at least equal to the disease output as an effect of the pandemic, expressed as a percentage of the base population of the analysed region.	We consider that the presented model should be adjusted, and the curve in the image depreciated with the value of the disturbing impact factors.
5.	Atkeson, A., 2020 [7]	The author, although correctly sensing the impact of the pandemic on the population’s health status and indirectly on the economy’s state as a whole, based on a model related to the Markov chains, is limited to using the scenario method only to quantify the effect of the pandemic over different exposure times, establishing a set of coefficients based on which the pandemic evolution curves are modelled.	The author’s conclusions support the need for economic analyses regarding the consequences of COVID-19 on the economy as a whole and on the public health segment. The proposed analysis is only an intermediate step in evaluating the general disturbing picture of economic growth.	From our point of view, our proposed model is more efficient and competitive and is able to quantify economic performance and to anticipate regional economic developments at least in the short term. The model can be improved by accumulating socio-economic influences on growth, which is also taken into account by our model.
6.	Gilchrist, S., Schoenle, R., Sim, J. and Zakrajsek, E., 2017 [8]	With relevant available data, the authors performed a pertinent analysis of the disturbing factors’ impacts, such as inflation of the global economy during the economic crisis. Thus, the authors correctly conclude that the financial disturbances influence the unjustified increase in the prices, having significant financial adverse effects on the stocks’ demand, affecting the liquidity and limiting the access to external financing. These factors act as markers of the growth curve’s flattening.	The model needs to be adjusted with the collateral effects of the need for financing due to the measures to combat the pandemic, and the need for financing due to social protection measures and economic recovery.	The issue of economic recovery financing is also treated by us through the prism of the investment process, which is affected by the allocations dedicated to the anti-pandemic fight, which is also reflected in our proposed model.
7.	Adrian, T., Fleming, M., Shachar, O. and Vogt, E., 2017 [9]	[9] The authors developed a detailed study of the financial market in the post-economic crisis period using data over a 25-year period, which included the economic crisis from 2007 to 2009. There was a disturbance on the financial markets within the financial crisis, able to change the trend of the capitalized assets, decelerating their growth under the impact of the financial crisis. The transactions’ volatility was presented as a peak during the crisis period, which subsequently tended to reach the values with a delay of 150% compared to the previous period. Thus, in order to calm the volatility, a period of 1.5 times greater than the period preceding the crisis is needed (6 years compared to 4 years). In this research, the debt security positions and the expected returns suffer a minor adjustment to the repayments of placements curve, which reinforces the concept of pessimism that the researchers have pointed out in the article. They also make a financial projection for 2 and 10 years of the effects of the economic crisis.	The model is a relevant one, which realistically captures the impact of the economic crisis on the global economy through the financial markets. The practical example is the situation in China since February 2020, when, in the midst of the economic crisis, the corporate bonds worth about 30% of their capitalized value were traded. These transactions were made in favour of the Chinese state.	We estimate that the occurrence of aggravating factors such as the COVID-19 pandemic and the exacerbation of financial consumption are able to amplify the pessimism of this model. The model can be improved by quantifying the social effect of the pandemic, as well as by quantifying the financial effect of fighting and preventing the disease, which directs a large proportion of economic resources to the medical field, leaving other economic areas uncovered and thus vulnerable (tourism, education, etc.).
8.	Kreichauf, R., 2018 [10]	The author analyses the phenomenon of refugee migration through a socio-spatial model elaborated in order to quantify the results of the measures of the refugees’ social inclusion and to accommodate them into the new socio-economic environment, including the quantification of the asylum austerities’ impact and the offered conditions in the refugee campuses in order to strengthen the norms of life safety on a sustainable basis.	There is pressure on asylum seekers that slows the social absorption of the asylum seekers and their integration into the new European socio-economic environment. The aspects invoked by the author must be assimilated into an impact study.	From our point of view, the socio-spatial model is not sufficient to achieve the research objectives proposed in the author’s study.
9.	Hangartner, D., Dinas, E., Marbach, M., Matakos, K. and Xefteris D., 2018 [11]	The authors conducted a study on the impact of the refugee crisis. The study was conducted on the basis of the information collected by the authors, modelled through the TSLS regression, observing, after modelling, the exacerbation of antisocial behaviour of the natives in the analysed territories in relation to migrants. The favouring factors of the normalized behavioural model are represented by the strengthening of border protection, the measures regarding the prevention of terrorist attacks and social protection measures. The study was conducted across 3 regions of Greece, Italy and Spain, of which Greece represents about 80% of the migrant waves.	The model aims to solve specific issues regarding the affectation of the native population by the migrants’ waves, on the basis of the cluster methodology. The unilateral approach is inferior to integrating information in a complex model based on the congruent evaluation of several disturbing factors with a long-term effect on the regional population.	In the current context of the crisis in Afghanistan, this model can be improved with the logistical and economic components which derive from the crisis situation created punctually by the withdrawal of troops from Afghanistan. This information can be a source for adjusting the indicators of the proposed model to predict the downstream and upstream economic dimension of migrant wave absorption.
10.	Harteveld, E., Schaper, J., De Lange, S.L. and Van Der Brug, W., 2017 [12]	The authors investigate how the refugee crisis affects the administrative capacity and the public opinion regarding the exercise of administrative attributes of governmental bodies on levels of influence. The Euroscepticism, as transpired from this study, was found to be directly proportional to the media phenomenon (the refugee crisis). The results of the study show that the polarization of the Europeans’ attitude in relation to this phenomenon of migration has the effect of lowering the support measures regarding the integration of new migrants into the European socio-economic life.	The authors analyse the dynamics of the mechanisms in relation to some secondary variables (for example, the media), which could have been replaced by an impact analysis of the socio-economic measures in relation to some primary aggravating factors such as those mentioned at the 9th point of Table 1.	In line with the current situation, we believe that improvements can be made both to the social and economic items as well as to the management and logistical strategy components of the refugee crisis.
11.	Danielli, S., Patria, R., Donnelly, P., Ashrafian, H., Darzi, A., 2020 [13]	The paper presented by the authors analyses the economic intervention to ameliorate the impact of COVID-19 on the economy and the health system through an international comparison, by which the European countries (Spain, Sweden, France, UK, Germany and Italy) appear with the most significant allocations of GDP in terms of fiscal measures in order to combat the effects of COVID-19. The structure of the package of measures (according to the authors) differs from state to state, with the caveat that tax cuts and the adoption of population support measures is an almost general pattern in the states analysed.	The authors manage to centralize some fiscal actions that may lead to certain action profiles during the pandemic, but the comparison between these profiles is weak. The results of the analysis can be further explored to draw more relevant conclusions.	The study is of interest, but needs to be deepened from the comparative analysis point of view as well as from the creating relevant conclusions point of view. In our study, we showed that some European countries (France, Spain, Italy and the UK) faced a significant economic impact, this rationale being the premise for developing working hypotheses leading to conclusive results in terms of regional performance under the impact of COVID-19.
12.	Umar, M., Xu, Y. and Mirza, S.S., 2021 [14]	The authors address the impact of the COVID-19 crisis on the labour market by analysing the impact of the pandemic on the GIG economy.	The results of the study show that, as far as the labour market is concerned, the degree of its affectation was in the closed economy area where many companies temporarily or permanently ceased their activity. At the same time, there have been online platforms where labour supply and demand could meet and generate a promising impact on “OLI filled jobs”.	Although it is an interesting study, the authors approach the effect of digitization on the labour market too optimistically, creating the premises for a positive effect of the transfer of labour supply from the real to the virtual environment. The analysis can be adjusted with additional correction factors on the economic contribution of telework productivity, even if some effects on pollution and social impact seem to confirm the hypotheses of the authors’ study. There are some sectors for which the positive impact can be quantified (services), but not in the productive sectors.
13.	Asahi K, Undurraga EA, Valdés R, Wagner R., 2021 [15]	The authors analyse in an interesting way the effect of COVID-19 on the economy in the context of lockdown. The method of analysis is the study of VAT collection in Chile during the lockdown and before the COVID-19 crisis in 170 municipalities.	The picture presented is relevant and proves that the pandemic has a profound disruptive effect on the affected economies. There is both a temporary and a geographical effect, with some regions having more manageable profiles than others.	We believe that the study can be improved by collecting the social components and the measures of prevention and control of the disease in order to highlight a general picture of action and effects during the pandemic period.
14.	Vitenu-Sackey, P.S. and Barfi, R., 2021 [16]	The authors analyse the global impact of the pandemic through a study in which they address both uncertainty and poverty alleviation in relation to economic growth. They point out that the COVID-19 pandemic far surpassed any other global pandemic produced between 1996 and 2020. The social component of the support measures reflects disparities in global economic development.	The model proposed by the authors’ aims to quantify the impact of the pandemic on the global economy and poverty alleviation. The model shows interesting composite variables such as the human development index (HDI) reported by the UN. The second composite indicator is the stringency index, which quantifies the impact of school closures, telecommuting and restrictions on free movement on society. These indicators in relation to GDP/capita and COVID’S monitoring indicators of illness and death are fed into a correlation matrix, showing that the pandemic is affecting economic growth and efforts to reduce the risk of poverty.	The study is topical, of interest and demonstrates the societal component as being of utmost importance in the pandemic equation.
15.	Khurshid, A. and Khan, K., 2020 [17]	The authors analyse the impact of COVID-19 on the environment and the economy, making projections up to 2032, based on dynamic modelling. The model takes into account the shock wave theory and is based on the indicators of energy consumption, population, GDP and climate change.	Long-term forecasting in an unpredictable global economy has little chance of verifying the forecasting model (“negative spike will decline the GDP by USD 6313.76 million in 2026”).	The scenario method may improve the conclusions of the study, which we find interesting. An increase in the number of indicators per economic segment can better substantiate the presented projections, increasing the reliability of the presentation.

**Table 2:** Model TSLS using observations 1–240. Dependent variable: C0; Instrumented: F1 F2 F3 F4; Instruments: C1 C2 C3 C4.

	Coefficient	Std. Error	t-Ratio	*p*-Value	
F1	2.18552	0.100009	21.85	<0.0001	***
F2	0.105132	0.0479492	2.193	0.0293	**
F3	0.0501419	0.0431048	1.163	0.2459	
F4	−1.33810	0.0780583	−17.14	<0.0001	***
Mean dependent var	3.150000	S.D. dependent var		1.150950	
The sum of the squares residuals	123.2029	Standard error of regression		0.722528	
Uncentred R-squared	0.728780	Centred R-squared		0.901506	
F (4.236)	1277.098	*p*-value(F)		1.5e^−158^	
Log-likelihood	−2566.499	Akaike criterion		5140.998	
Schwarz criterion	5154.920	Hannan–Quinn		5146.608	

***—high significance; **—medium significance.

## Data Availability

We used public data from Eurostat and the World Health Organization.

## Appendix A

Distribution of the scalar values of the disturbing factors across the Member States, and their impact on regional administrative capacity.

## Appendix B

**Table A1:** COVID-19’s impact on NUTS 2 regions.

Regions	COVID-19 Region Cases	Population on 1 January 2019 by NUTS 2 Region	COVID-19 Rate	COVID-19 Scalar	Regions	COVID-19 Region Cases	Population on 1 January 2019 by NUTS 2 Region	COVID-19 Rate	COVID-19 Scalar
Région de Bruxelles/Brussels	2352	1,215,290	0.19%	1	Languedoc-Roussillon	4150	2,838,966	0.15%	1
Antwerpen	363	1,860,470	0.02%	3	Midi-Pyrénées	4480	3,060,243	0.15%	1
Limburg	0	875,842	0.00%	5	Auvergne	1990	1,362,576	0.15%	1
Oost-Vlaanderen	0	1,516,283	0.00%	5	Rhône-Alpes	5720	6,643,306	0.09%	3
Vlaams-Brabant	0	1,146,643	0.00%	5	Provence-Alpes-Côte d’Azur	7380	5,048,405	0.15%	1
West-Vlaanderen	0	1,196,995	0.00%	5	Corse	225	341,554	0.07%	3
Brabant wallon	5809	404,270	1.44%	1	Guadeloupe	76	416,474	0.02%	3
Hainaut	0	1,346,082	0.00%	5	Martinique	66	363,484	0.02%	3
Liège	12,289	1,110,068	1.11%	1	Guyane	28	283,539	0.01%	4
Luxembourg	0	286,685	0.00%	5	La Réunion	94	857,961	0.01%	4
Namur	0	496,891	0.00%	5	Mayotte	35	269,471	0.01%	4
Severozapaden	588	742,304	0.08%	3	Jadranska Hrvatska	611	1,374,071	0.04%	3
Severen tsentralen	0	784,168	0.00%	5	Kontinentalna Hrvatska	671	2,702,175	0.02%	3
Severoiztochen	0	929,035	0.00%	5	Piemonte	13,343	4,356,406	0.31%	1
Yugoiztochen	0	1,032,079	0.00%	5	Valle d’Aosta	835	125,666	0.66%	1
Yugozapaden	0	2,102,205	0.00%	5	Liguria	4757	1,550,640	0.31%	1
Yuzhen tsentralen	0	1,410,248	0.00%	5	Lombardia	52,325	10,060,574	0.52%	1
Praha	1234	1,308,632	0.09%	3	Provincia Autonoma di Bolzano	1811	531,178	0.34%	1
Strední Cechy	606	1,369,332	0.04%	3	Provincia Autonoma di Trento	2476	541,098	0.46%	1
Jihozápad	298	1,226,805	0.02%	3	Veneto	11,925	4,905,854	0.24%	1
Severozápad	278	1,115,685	0.02%	3	Friuli-Venezia Giulia	2153	1,215,220	0.18%	1
Severovýchod	1300	1,513,693	0.09%	3	Emilia-Romagna	17,825	4,459,477	0.40%	1
Jihovýchod	334	1,696,941	0.02%	3	Toscana	6173	3,729,641	0.17%	1
Strední Morava	450	1,215,413	0.04%	3	Umbria	1263	882,015	0.14%	2
Moravskoslezsko	506	1,203,299	0.04%	3	Marche	4710	1,525,271	0.31%	1
Hovedstaden	1901	1,835,562	0.10%	3	Lazio	4149	5,879,082	0.07%	3
Sjælland	203	836,738	0.02%	3	Abruzzo	1799	1,311,580	0.14%	2
Syddanmark	472	1,223,348	0.04%	3	Molise	224	305,617	0.07%	3
Midtjylland	584	1,320,678	0.04%	3	Campania	3148	5,801,692	0.05%	3
Nordjylland	501	589,755	0.08%	3	Puglia	2514	4,029,053	0.06%	3
Stuttgart	3189	4,143,418	0.08%	3	Basilicata	291	562,869	0.05%	3
Karlsruhe	2234	2,805,129	0.08%	3	Calabria	833	1,947,131	0.04%	3
Freiburg	2084	2,264,469	0.09%	3	Sicilia	2097	4,999,891	0.04%	3
Tübingen	7432	1,856,517	0.40%	1	Sardegna	935	1,639,591	0.06%	3
Oberbayern	2123	4,686,163	0.05%	3	Kypros	494	875,899	0.06%	3
Niederbayern	3112	1,238,528	0.25%	1	Latvija	548	1,919,968	0.03%	3
Oberpfalz	4311	1,109,269	0.39%	1	Sostines regionas	412	810,538	0.05%	3
Oberfranken	2120	1,067,482	0.20%	1	Vidurio ir vakaru Lietuvos regionas	468	1,983,646	0.02%	3
Mittelfranken	1234	1,770,401	0.07%	3	Luxembourg	2970	613,894	0.48%	1
Unterfranken	3214	1,317,124	0.24%	1	Budapest	345	1,752,286	0.02%	3
Schwaben	1245	1,887,754	0.07%	3	Pest	153	1,278,874	0.01%	4
Berlin	3670	3,644,826	0.10%	3	Közép-Dunántúl	139	1,058,236	0.01%	4
Brandenburg	2345	2,511,917	0.09%	3	Nyugat-Dunántúl	86	989,343	0.01%	4
Bremen	2811	682,986	0.41%	1	Dél-Dunántúl	4	879,596	0.00%	5
Hamburg	2993	1,841,179	0.16%	1	Észak-Magyarország	82	1,126,360	0.01%	4
Darmstadt	1121	3,998,724	0.03%	3	Észak-Alföld	33	1,450,960	0.00%	5
Gießen	1125	1,047,262	0.11%	3	Dél-Alföld	70	1,237,101	0.01%	4
Kassel	1889	1,219,823	0.15%	1	Malta	293	493,559	0.06%	3
Mecklenburg-Vorpommern	2346	1,609,675	0.15%	1	Groningen	207	583,990	0.04%	3
Braunschweig	1256	1,596,396	0.08%	3	Friesland	215	647,672	0.03%	3
Hannover	1084	2,149,805	0.05%	3	Drenthe	222	492,167	0.05%	3
Lüneburg	1111	1,710,914	0.06%	3	Overijssel	1211	1,156,431	0.10%	3
Weser-Ems	3456	2,525,333	0.14%	2	Gelderland	2210	2,071,972	0.11%	3
Düsseldorf	2412	5,202,321	0.05%	3	Flevoland	264	416,546	0.06%	3
Köln	1525	4,468,904	0.03%	3	Utrecht	1476	1,306,912	0.11%	3
Münster	3500	2,623,619	0.13%	3	Noord-Holland	2891	2,853,359	0.10%	3
Detmold	2235	2,055,310	0.11%	3	Zuid-Holland	3361	3,709,139	0.09%	3
Arnsberg	2768	3,582,497	0.08%	3	Zeeland	279	383,032	0.07%	3
Koblenz	2987	1,495,885	0.20%	1	Noord-Brabant	4456	2,544,806	0.18%	1
Trier	3456	531,007	0.65%	1	Limburg	2011	1,116,137	0.18%	1
Rheinhessen-Pfalz	1567	2,057,952	0.08%	3	Burgenland	234	293,433	0.08%	3
Saarland	4321	990,509	0.44%	1	Niederösterreich	1978	1,677,542	0.12%	3
Dresden	3189	1,598,199	0.20%	1	Wien	1777	1,897,491	0.09%	3
Chemnitz	4567	1,436,445	0.32%	1	Kärnten	333	560,939	0.06%	3
Leipzig	3111	1,043,293	0.30%	1	Steiermark	1354	1,243,052	0.11%	3
Sachsen-Anhalt	2345	2,208,321	0.11%	3	Oberösterreich	2061	1,482,095	0.14%	2
Schleswig-Holstein	2723	2,896,712	0.09%	3	Salzburg	1085	555,221	0.20%	1
Thüringen	3456	2,143,145	0.16%	1	Tirol	2804	754,705	0.37%	1
Eesti	1149	1,324,820	0.09%	3	Vorarlberg	764	394,297	0.19%	1
Northern and Western	1389	867,947	0.16%	1	Malopolskie	1247	3,360,545	0.04%	3
Southern	2510	1,624,381	0.15%	1	Slaskie	601	4,488,998	0.01%	4
Eastern and Midland	1810	2,411,912	0.08%	3	Wielkopolskie	349	3,473,172	0.01%	4
Anatoliki Makedonia, Thraki	367	599,723	0.06%	3	Zachodniopomorskie	133	1,675,502	0.01%	4
Kentriki Makedonia	215	1,873,777	0.01%	4	Lubuskie	127	1,003,310	0.01%	4
Dytiki Makedonia	314	267,008	0.12%	3	Dolnoslaskie	527	2,865,072	0.02%	3
Ipeiros	98	333,696	0.03%	3	Opolskie	107	946,038	0.01%	4
Thessalia	412	718,640	0.06%	3	Kujawsko-Pomorskie	264	2,055,433	0.01%	4
Ionia Nisia	190	203,869	0.09%	3	Warminsko-Mazurskie	87	1,404,441	0.01%	4
Dytiki Ellada	78	655,189	0.01%	4	Pomorskie	135	2,305,077	0.01%	4
Sterea Ellada	58	555,960	0.01%	4	Lódzkie	358	2,453,167	0.01%	4
Peloponnisos	70	574,447	0.01%	4	Swietokrzyskie	120	1,226,243	0.01%	4
Attiki	12	3,742,235	0.00%	5	Lubelskie	192	2,097,294	0.01%	4
Voreio Aigaio	15	221,098	0.01%	4	Podkarpackie	371	2,086,135	0.02%	3
Notio Aigaio	0	344,027	0.00%	5	Podlaskie	168	1,152,074	0.01%	4
Kriti	3	634,930	0.00%	5	Warszawski stoleczny	62	3,053,104	0.00%	5
Galicia	6331	2,700,441	0.23%	1	Mazowiecki regionalny	0	2,327,207	0.00%	5
Principado de Asturias	1679	1,022,205	0.16%	1	Norte	1777	3,572,583	0.05%	3
Cantabria	1501	581,641	0.26%	1	Algarve	1561	438,864	0.36%	1
País Vasco	9021	2,177,880	0.41%	1	Centro	1823	2,216,569	0.08%	3
Navarra	3355	649,946	0.52%	1	Lisboa	1789	2,846,332	0.06%	3
La Rioja	2846	313,571	0.91%	1	Alentejo	1813	705,478	0.26%	1
Aragón	3449	1,320,586	0.26%	1	Região Autónoma dos Açores	1800	242,846	0.74%	1
Madrid	40,469	6,641,649	0.61%	1	Madeira	1879	253,945	0.74%	1
Castilla y León	9581	2,407,733	0.40%	1	Nord-Vest	276	2,552,112	0.01%	4
Castilla-la Mancha	11,077	2,034,877	0.54%	1	Centru	425	2,318,272	0.02%	3
Extremadura	2116	1,065,424	0.20%	1	Nord-Est	1743	3,198,564	0.05%	3
Cataluña	28,323	7,566,431	0.37%	1	Sud-Est	362	2,396,171	0.02%	3
Comunidad Valenciana	7443	4,974,969	0.15%	1	Sud-Muntenia	282	2,929,832	0.01%	4
Illes Balears	1369	1,188,220	0.12%	3	Bucuresti-Ilfov	697	2,315,173	0.03%	3
Andalucía	8767	8,427,405	0.10%	3	Sud-Vest Oltenia	72	1,926,860	0.00%	5
Región de Murcia	1283	1,487,663	0.09%	3	Vest	565	1,777,474	0.03%	3
Ciudad Autónoma de Ceuta	83	84,829	0.10%	3	Vzhodna Slovenija	501	1,094,435	0.05%	3
Ciudad Autónoma de Melilla	92	84,689	0.11%	3	Zahodna Slovenija	558	986,473	0.06%	3
Canarias	1725	2,206,901	0.08%	3	Bratislavský kraj	147	659,598	0.02%	3
Île de France	17,910	12,244,807	0.15%	1	Západné Slovensko	76	1,826,145	0.00%	5
Centre-Val de Loire	3750	2,565,258	0.15%	1	Stredné Slovensko	72	1,339,242	0.01%	4
Bourgogne	1569	1,619,728	0.10%	3	Východné Slovensko	286	1,625,436	0.02%	3
Franche-Comté	24,920	1,173,605	2.12%	1	Länsi-Suomi	90	1,379,749	0.01%	4
Basse-Normandie	688	1,464,500	0.05%	3	Helsinki	1438	1,671,024	0.09%	3
Haute-Normandie	1753	1,848,840	0.09%	3	Etelä-Suomi	154	1,152,719	0.01%	4
Nord-Pas-de-Calais	5930	4,052,299	0.15%	1	Pohjois- ja Itä-Suomi	118	1,284,638	0.01%	4
Picardie	2820	1,925,138	0.15%	1	Åland	508	29,789	1.71%	1
Alsace	2770	1,894,144	0.15%	1	Stockholm	3279	2,344,124	0.14%	2
Champagne-Ardenne	1920	1,314,964	0.15%	1	Östra Mellansverige	1133	1,708,813	0.07%	3
Lorraine	3390	2,316,183	0.15%	1	Småland med öarna	632	864,630	0.07%	3
Pays-de-la-Loire	368	3,787,400	0.01%	4	Sydsverige	366	1,521,848	0.02%	3
Bretagne	603	3,333,720	0.02%	3	Västsverige	674	2,039,166	0.03%	3
Aquitaine	1082	3,450,045	0.03%	3	Norra Mellansverige	554	855,220	0.06%	3
Limousin	1070	729,981	0.15%	1	Mellersta Norrland	576	375,733	0.15%	1
Poitou-Charentes	2640	1,806,292	0.15%	1	Övre Norrland	478	520,651	0.09%	3
